# P-1277. Carbapenemase-producing Enterobacterales (CPE): Specimen Testing, Enzyme Comparison, Timeline Analysis and Patient Outcomes at a large tertiary UK centre

**DOI:** 10.1093/ofid/ofaf695.1467

**Published:** 2026-01-11

**Authors:** Lee Xin Ting, Neil Nathwani, Itisha Gupta

**Affiliations:** University Hospitals Birmingham NHS Foundation Trust, United Kingdom, Birmingham, England, United Kingdom; University Hospitals Birmingham NHS Foundation Trust, United Kingdom, Birmingham, England, United Kingdom; UK Health Security Agency (UKHSA) Birmingham, United Kingdom, Birmingham, England, United Kingdom

## Abstract

**Background:**

Carbapenemase-producing Enterobacterales (CPE) is a growing healthcare concern, requiring multidisciplinary efforts and significant resources. Screening guidelines in the UK specify criteria for testing and confirmation.

**Figure 1.**
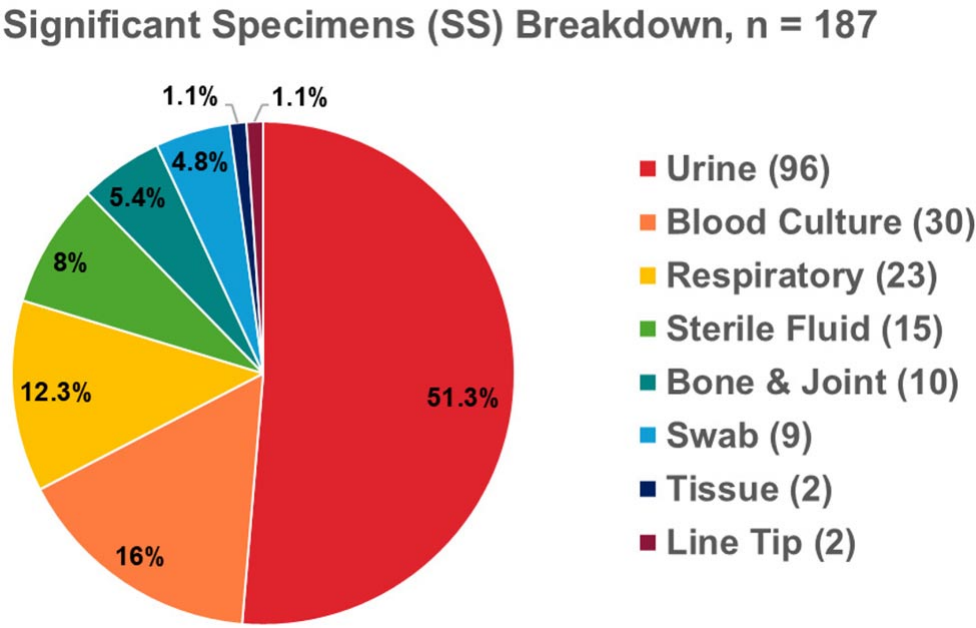
illustrates the breakdown of positive significant specimens (SS) included within our study across the 6-year period (January 2019 - December 2024), with predominant sites being urine (51.3%), blood culture (16%) and respiratory (12.3%).

**Figure 2.**
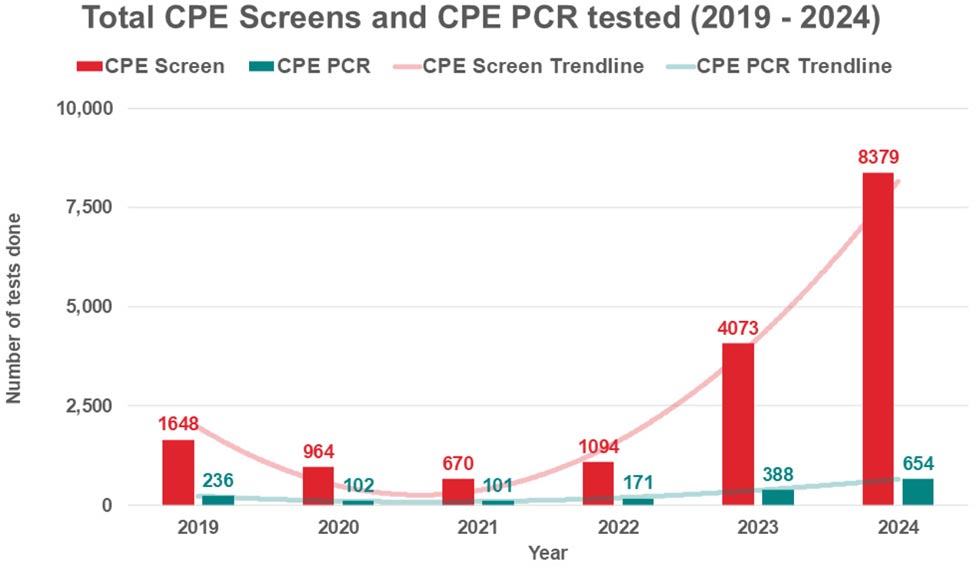
illustrates the total numbers of CPE screens and CPE PCRs tested within our centre across the 6-year period (January 2019 - December 2024), demonstrating accelerated rise from 2021-2024. CPE screening was conducted using selective chromogenic culture media for screen specimens, primarily rectal swabs. For significant specimens (SS), carbapenem resistance was picked up from routine direct disc susceptibility testing (ertapenem and meropenem) alongside the bioMérieux VITEK® susceptibility testing platform. All specimens were then confirmed via CPE PCR, or presumed positive in the presence of a recent CPE PCR positive specimen of the same species within the patient's timeline, by means of leveraging existing data to minimize redundant testing,

**Methods:**

Specimen data (Jan 2019-Dec 2024) across three hospital sites were retrospectively analysed for CPE positivity, based on prior or subsequent positive confirmation testing via polymerase chain reaction (PCR - NDM-1, KPC, VIM, OXA-48, IMP) and/ or carbapenem e-tests. Specimens were divided into screens and significant specimens (SS), with different species within a single test regarded separately. Patient demographics and clinical outcomes were reviewed.

**Figure 3.**
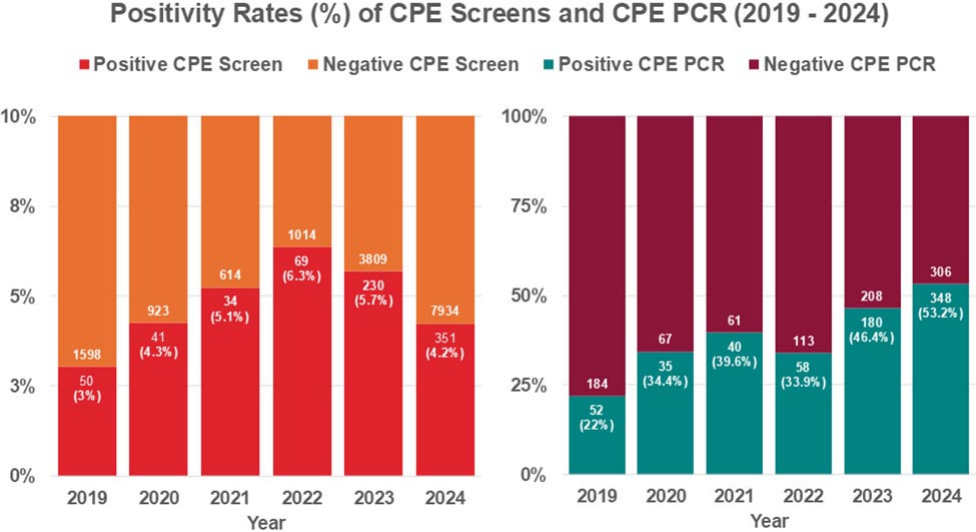
illustrates the number of positive and negative CPE screens and CPE PCRs recorded within our centre across the 6-year period (January 2019 - December 2024), with positivity rates for each year listed under the number of positive results within closed brackets.

**Figure 4.**
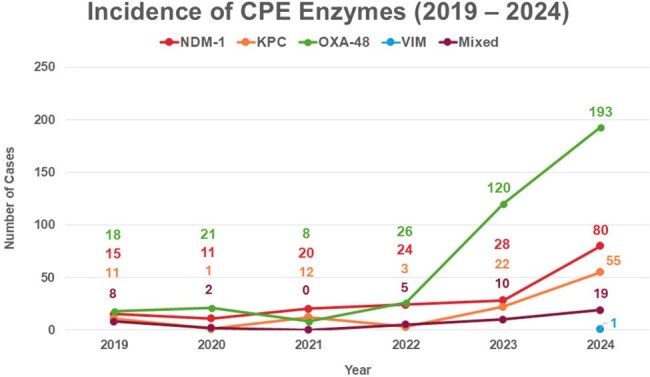
illustrates the incidence of CPE-positive enzymes within our centre across the 6-year period (January 2019 - December 2024), with predominant enzymes being OXA-48, NDM-1 and KPC. No cases of IMP was recorded, with only 1 case of VIM out of 962 total specimens.

**Results:**

Of 962 CPE-positive specimens (80.6% screens, 19.4% SS), 73.2% of screens and 78.1% of SS had PCR confirmation. Overall testing increased from 2021-2024, with screening positivity peaking at 6.3% (2022) then falling to 4.2%, while PCR positivity rose to 53.2% (2024). Main species were *Escherichia coli* (46.2%), *Klebsiella pneumoniae* (26.7%) and *Enterobacter cloacae* (19.4%).

Key enzymes were OXA-48 (54.1%), NDM-1 (25%), KPC (14.6%) and mixed (6.2%, mostly NDM-1+OXA-48). Resistance rates were generally high for **β**-lactams, but low for tigecycline, nitrofurantoin and fosfomycin. NDM-1 showed higher overall resistance, including to cefiderocol and newer combinations. Mixed (34.4%) and KPC (15.5%) screens progressed to SS more than NDM-1 (7.4%) and OXA-48 (7.9%).

Meanwhile, SS with prior positive screens were most often mixed enzymes (58.3%), then OXA-48 (37.9%), NDM-1 (33.3%) and KPC (26.1%). Within this group's timeline, species was consistent in 93.6% and enzyme group in 88.2%. Median time from first positive screen to SS was 37 days, with a median of 2 prior positive screens.

Among 454 patients (56.2% male, median age 75), all-cause mortality was 12.1% at 30-day, 18.3% at 90-day and 29.1% at 1-year. Mixed and KPC enzymes had higher ICU admission rates and longer inpatient stay.

**Conclusion:**

Our study highlights the increasing burden of CPE screening, diagnosis and management, with notable inter-enzyme differences in incidence, antimicrobial resistance, progression to infection and patient outcomes. Ongoing surveillance and tailored strategies are vital to combat this global threat.

**Disclosures:**

All Authors: No reported disclosures

